# The breast cancer susceptibility-related polymorphisms at the TOX3/LOC643714 locus associated with lung cancer risk in a Han Chinese population

**DOI:** 10.18632/oncotarget.10874

**Published:** 2016-07-28

**Authors:** Chaowen Jiang, Shilong Yu, Pin Qian, Ruiling Guo, Ruijie Zhang, Zhi Ao, Qi Li, Guoming Wu, Yan Chen, Jin Li, Changzheng Wang, Wei Yao, Jiancheng Xu, Guisheng Qian, Fuyun Ji

**Affiliations:** ^1^ Institute of Human Respiratory Disease, Xinqiao Hospital, The Third Military Medical University, Chongqing 400037, China; ^2^ Department of Respiratory Diseases, 324^th^ Hospital of People's Liberation Army (No.324 Hospital of PLA), Chongqing 400020, China; ^3^ Department of Respiratory Medicine, The Third Xiangya Hospital, Central South University, Changsha, Hunan 410013, China

**Keywords:** lung cancer, risk, TOX3/LOC643714, polymorphisms, breast cancer

## Abstract

It has been well established that besides environmental factors, genetic factors are also associated with lung cancer risk. However, to date, the prior identified genetic variants and loci only explain a small fraction of the familial risk of lung cancer. Hence it is vital to investigate the remaining missing heritability to understand the development and process of lung cancer. In the study, to test our hypothesis that the previously identified breast cancer risk-associated genetic polymorphisms at the TOX3/LOC643714 locus might contribute to lung cancer risk, 16 SNPs at the TOX3/LOC643714 locus were evaluated in a Han Chinese population based on a case-control study. Pearson's chi-square test or Fisher's exact test revealed that rs9933638, rs12443621, and rs3104746 were significantly associated with lung cancer risk (*P* < 0.001, *P* < 0.001, and *P* = 0.005, respectively). Logistic regression analyses displayed that lung cancer risk of individuals with rs9933638(GG+GA) were 1.89 times higher than that of rs9933638AA carriers (OR = 1.893, 95% CI = 1.308-2.741, *P* = 0.001). Similar findings were manifested for rs12443621 (OR = 1.824, 95% CI = 1.272-2.616, *P* = 0.001, rs12443621(GG+GA) carriers vs. rs12443621AA carriers) and rs3104746 (OR = 1.665, 95% CI = 1.243-2.230, *P* = 0.001, rs3104746TT carriers vs. rs3104746(TA+AA) carriers). The study discovered for the first time that three SNPs (rs9933638, rs12443621, and rs3104746) at the TOX3/LOC643714 locus contributed to lung cancer risk, providing new evidences that lung cancer and breast cancer are linked at the molecular and genetic level to a certain extent.

## INTRODUCTION

Lung cancer is a major public health concern worldwide, causing as many deaths as next four most deadly cancers combined (breast, prostate, colon, and pancreas). Non-small cell lung cancer (NSCLC) is the commonest lung cancer histology, comprised primarily of adenocarcinoma and squamous cell carcinoma [1]. In China, lung cancer has replaced liver cancer to become the leading cause of cancer-related deaths and accounted for 29% of all male cancer deaths and 23% of all female cancer deaths, totally approximately 220,5200 deaths, in 2014 (World Health Statistics, WHO, 2014). It has been established that multiple environment (mainly cigarette smoking and asbestos) and genetic factors are involved and gene-environment interactions exist in the development and process of lung cancer [2–4].

In the past few years, several genetic variants and loci have been identified to be the genetic risk factors of lung cancer [5–9]. However, to date, these known common loci only explain a small fraction of the familial risk of lung cancer. Hence it is vital to investigate the remaining missing heritability to understand the development and process of lung cancer. The TOX3/LOC643714 locus on chromosome 16q12.1 was one of the first breast cancer regions identified through genome-wide association study (GWAS) in populations of European and East Asian origin [10]. The chromosomal region spanning the 5′ end of TOX3, the intergenic region between TOX3 and LOC643714, and the entire coding part of LOC643714 are located in a 133 kb linkage disequilibrium (LD) block [11]. LOC643714 is an uncharacterized gene of unknown function [http://www.ncbi.nlm.nih.gov/gene/643714]. Identified first in a screen for transcripts containing trinucleotide repeat expansions, TOX3 gene, also termed trinucleotide repeat containing 9 (TNRC9), belongs to the high-mobility group (HMG) family of nonhistone chromatin proteins, indicating its potential role as a transcript factor [12] and involvement in bending and unwinding of DNA and alteration of chromatin structure [13]. TOX3 is largely expressed within the brain in the frontal and occipital lobe, the central nervous system (CNS), and the ileum in normal human tissues. Through interaction with the cAMP-response-element-binding protein (CREB), TOX3 regulates Ca^2+^-dependent neuronal transcription [14]. The overexpression of TOX3 induces transcription involving isolated estrogen responsive elements and estrogen-responsive promoters, and protects neuronal cells from cell death caused by endoplasmic reticulum stress or BCL2-Associated X Protein (BAX) overexpression through the induction of anti-apoptotic transcripts and repression of pro-apoptotic transcripts [15].

In the several GWAS, a number of novel genetic variants and loci at the TOX3/LOC643714 locus were identified to be independently associated with elevated risk of breast cancer and rs3803662 was highlighted for its potential biological contribution to the development of breast cancer. Rs3803662 was first reported to be related with breast cancer risk in an Icelandic population [13]. The following studies demonstrated that rs3803662 was not only associated with increased risk of breast cancer in both BRCA1 and BRCA2-mutation carriers and estrogen receptor (ER) positive patients [16], male breast cancer [17, 18], triple-negative (TN) breast cancer [19, 20], but also with overall survival (OS) of breast cancer [21, 22]. The other SNPs at the TOX3/LOC643714 locus reported to be associated with risk of breast cancer included rs3104746 [23, 24], rs8051542 [25–30], rs4784227 [25, 31–34], rs12443621 [25–27, 35–37], rs3112612 [25, 38], rs3112562 [23], rs3104793 [23, 39], and rs8046994 [23]. Among them, a slice of SNPs were shown to be related with risk of breast cancer in certain ethnic populations. For example, rs3104746, rs3112562, rs3104793, and rs8046994 were indicated to be associated with risk of breast cancer in an African-American women population [23]. Rs8051542 was found to be significantly associated with breast cancer risk in Tunisians [27] and rs3112612 yielded moderate predictive power in Ashkenazi Jewish women with strong family histories but no identifiable BRCA1/2 mutation [37]. Moreover, rs8051542, rs3803662, rs4784227, and rs12443621 were found to be associated with breast cancer risk of Chinese population [25, 28, 37]. Besides breast cancer, rs3104788 and rs3104767 were indicated to be susceptible to periodic leg movements (PLM) [40, 41] and restless legs syndrome susceptibility [42, 43], respectively.

Though the known etiology and carcinogenesis of lung cancer are different from that of breast cancer, the patients of the two diseases could be treated by some common chemotherapeutic agents such as taxanes (paclitaxel and taxotere), vincristine (Navelbine, NVB), and platinum-containing anticancer drugs (cisplatin and carboplatin). Most importantly and interestingly, a recent meta-analysis based on four lung cancer GWAS in populations of European ancestry, the MD Anderson Cancer Center (MDACC) GWAS, the Institute of Cancer Research (ICR) GWAS, the National Cancer Institute (NCI) GWAS, and the International Agency for Research on Cancer (IARC) GWAS, identified a rare variant of *BRCA2* gene, a well-known risk factor for breast, ovarian, and aggressive prostate cancers, to an increased risk of squamous cell lung cancer among cigarette smokers, suggesting that lung cancer and breast cancer are linked at a molecular and genetic level to a certain extent [44].

Due to the findings of a molecular and genetic linkage between lung cancer and breast cancer and the potential involvement of TOX3 in bending and unwinding of DNA and alteration of chromatin structure, we deduced that the previously identified breast cancer susceptibility-associated variants and loci at the TOX3/LOC643714 locus may contribute to lung cancer risk. To test the hypothesis, 16 SNPs at the TOX3/LOC643714 locus were selected and genotyped in a Han Chinese population from Southwestern China based on a case-control study. The genotyping data demonstrated that three SNPs (rs9933638, rs12443621, and rs3104746) at the TOX3/LOC643714 locus were associated with elevated risk of lung cancer and might be potentially biologically relevant to lung carcinogenesis.

## RESULTS

### Subject characteristics

Totally, 352 unrelated patients and 407 unrelated controls were recruited from Southwestern China for the case-control study. No female cigarette smokers were gathered. The general descriptive characteristics of the study population were given in Table 1. The median number of pack-years of combined cases and controls was utilized as the cut-point to stratify the cigarette smoking subjects. As shown in Table 1, there were no significant difference in gender and age between the controls and cases. As expected, cases smoked more cigarettes (*P*< 0.001). The distribution of tumour types among the patients was as follows: adenocarcinoma, 42.05%; squamous cell carcinoma, 28.13%; other non-small cell carcinoma, 16.19%; and small cell carcinoma, 13.64%.

**Table 1:** General characteristics of controls and lung cancer cases in a Han Chinese population

Characteristics	Controls (n = 407) (%)	Cases (n = 352) (%)	*p*-Value
Gender			
Male	320 (78.62)	272 (77.27)	0.654^a^
Female	87(21.38)	80 (22.73)	
Age at diagnosis (years)			
<39	23 (5.65)	18(5.11)	0.819^a^
40-49	73(17.94)	54 (15.34)	
50-59	125 (30.71)	112 (31.82)	
60-69	116 (28.50)	104 (29.55)	
>70	70 (17.20)	63 (17.90)	
Mean age ± SD	57.90 ±10.01	58.88 ±11.40	0.916^b^
Histology			
Adenocarcinoma	-	148 (42.05)	-
Squamous cell carcinoma	-	99 (28.13)	
Other non-small cell carcinoma	-	57 (16.19)	
Small cell carcinoma	-	48 (13.64)	
Pack-years of smoking^c^			
0-30	359 (88.21)	228 (64.77)	0.0001^a^
>30	48 (11.79)	124 (35.23)	
Mean pack-year ± SD	8.07 ± 16.65	22.44 ±19.53	0.008b
Mean pack-year ± SD^d^	12.04 ± 20.81	31.63 ± 21.13	0.001b

a *χ*^2^-Test or Fisher's exact test.

b *t*-Test.

c The median number of pack years of combined cases and controls were utilized as the cut-point.

d Restricted to males only.

### Association of the alleles of the 16 SNPs with lung cancer risk

The basic information regarding the 16 SNPs at the TOX3/LOC643714 locus was demonstrated in Supplementary Table S3. The 16 SNPs were genotyped in all of the lung cancer patients and healthy controls and qualified according to Hardy-Weinberg equilibrium (HWE) in the study population (Supplementary Table S3). As shown in Table 2, Pearson's chi-square test or Fisher's exact test demonstrated that of the 16 SNPs, three SNPs (rs9933638, rs12443621, and rs3104746) were found to be significantly associated with lung cancer risk (*P* < 0.001, *P* < 0.001, and *P* = 0.005, respectively) and rs3095661 displayed a marginally significance (*P* = 0.041) (Table 2). Among the four SNPs, rs9933638, representing a haplotype block covering 12 SNPs including rs12443621, is located at intron 2 of TOX3. Rs3095661 and rs3104746 are located at intron 4 of TOX3 and intron 2 of LOC643714, respectively (Supplementary Table S3).

**Table 2:** Distribution of the alleles of the 16 SNPs at the TOX3/LOC643714 locus between controls and cases in the study

SNPs	Allele	Control (n=407)(%)	Lung cancer (n=352)(%)	*P* Value^a^	Adeno- carcinoma (n=148)(%)	*P* Value^a^	Squamous cell carcinoma (n=99)(%)	*P* Value^a^	Male			Female		
									Control (n=320)(%)	Lung cancer (n=272)(%)	*P* Value^a^	Control (n=87)(%)	Lung cancer (n=80)(%)	*P* Value^a^
rs3095661	G	728(89.43)	605(85.94)	0.041	247(83.45)	0.009	182(92.11)	0.357	573(89.53)	469(86.21)	0.088	155(89.08)	136(85.00)	0.327
	C	86(10.57)	99(14.06)		49(16.55)		16(7.89)		67(10.47)	75(13.79)		19(10.92)	24(15.00)	
rs10852413	T	665(81.70)	570(81.20)	0.842	237(80.07)	0.543	163(82.32)	0.918	520(81.25)	444(81.92)	0.821	145(83.33)	126(78.75)	0.328
	C	149(18.30)	132(18.80)		59(19.92)		35(17.68)		120(18.75)	98(18.08)		29(16.67)	34(21.25)	
rs16951204	G	536(65.85)	485(68.89)	0.228	186(62.84)	0.356	131(66.16)	1.000	418(65.31)	380(69.85)	0.106	118(67.82)	105(65.63)	0.728
	C	278(34.15)	219(31.11)		110(37.16)		67(33.84)		222(34.69)	164(30.15)		56(32.18)	55(34.38)	
rs4784219	A	595(73.10)	523(74.50)	0.559	229(77.37)	0.163	149(75.25)	0.590	469(73.28)	402(74.17)	0.741	126(72.41)	121(75.63)	0.534
	T	219(26.90)	179(25.50)		67(22.63)		49(24.75)		171(26.72)	140(25.83)		48(27.59)	39(24.38)	
rs9302555	T	630(77.40)	557(79.12)	0.455	241(81.42)	0.161	159(80.26)	0.391	500(78.13)	445(81.80)	0.652	130(74.71)	112(70.00)	0.391
	G	184(22.60)	147(20.88)		55(18.58)		39(19.74)		140(21.88)	99(18.20)		44(25.29)	48(30.00)	
rs8051542	C	657(83.38)	577(81.96)	0.493	247(83.33)	1.000	177(89.47)	0.803	519(83.57)	442(81.25)	0.316	138(82.63)	135(84.38)	0.766
	T	131(16.62)	127(18.04)		49(16.67)		21(10.53)		102(16.43)	102(18.75)		29(17.37)	25(15.62)	
rs9933638	G	445(54.80)	465(66.05)	0.000	200(67.57)	0.0001	130(65.79)	0.006	352(55.17)	356(65.44)	0.000	93(53.45)	109(68.13)	0.007
	A	367(45.20)	239(33.95)		96(32.43)		68(34.21)		286(44.83)	188(34.563)		81(46.55)	51(31.88)	
rs12443621	G	450(55.28)	459(65.20)	0.000	197(66.67)	0.0007	128(64.47)	0.020	358(55.94)	350(64.34)	0.003	92(52.87)	109(68.13)	0.005
	A	364(44.72)	245(34.80)		99(33.33)		70(35.53)		282(44.06)	194(35.66)		82(47.13)	51(31.88)	
rs3095604	C	556(68.30)	470(66.76)	0.545	200(67.57)	0.827	128(64.42)	0.352	430(67.19)	368(67.65)	0.901	126(72.41)	102(63.75)	0.100
	G	258(31.70)	234(33.24)		96(32.43)		70(35.53)		210(32.81)	176(32.35)		48(27.59)	58(36.25)	
rs1362550	G	652(80.10)	576(81.82)	0.432	244(82.43)	0.439	151(76.32)	0.241	506(79.06)	440(83.91)	0.467	146(83.91)	136(85.00)	0.880
	C	162(19.90)	128(18.18)		52(17.57)		47(23.68)		134(20.94)	104(16.09)		28(16.09)	24(15.00)	
rs28463809	T	558(68.55)	466(66.19)	0.351	194(65.54)	0.346	128(64.47)	0.309	439(68.59)	368(67.65)	0.754	119(68.39)	98(61.25)	0.201
	G	256(31.45)	238(33.81)		102(34.46)		70(35.53)		201(31.41)	176(32.35)		55(31.61)	62(38.75)	
rs4784226	C	616(75.68)	527(74.86)	0.721	220(74.32)	0.638	154(75.63)	0.578	488(76.25)	406(74.63)	0.542	128(73.56)	121(75.63)	0.707
	T	198(24.32)	177(25.14)		76(25.68)		44(22.37)		152(23.75)	138(25.37)		46(26.44)	39(24.38)	
rs3803662	A	550(67.57)	472(67.05)	0.869	198(66.89)	0.829	128(64.47)	0.449	426(67.30)	370(68.01)	0.619	124(71.26)	102(63.75)	0.161
	G	264(32.43)	232(32.95)		98(31.11)		70(35.53)		214(32.70)	174(31.99)		50(28.74)	58(36.25)	
rs4784227	C	616(75.68)	530(75.28)	0.905	220(74.44)	0.638	156(78.95)	0.402	488(76.25)	408(75.00)	0.635	128(73.56)	122(76.25)	0.615
	T	198(24.32)	174(24.72)		76(25.56)		42(21.05)		152(23.75)	136(25.00)		46(26.44)	38(23.75)	
rs3104746	T	665(81.70)	613(87.07)	0.005	257(86.67)	0.047	177(89.39)	0.008	525(82.03)	471(86.58)	0.038	140(80.46)	142(88.75)	0.049
	A	149(18.30)	91(12.93)		39(13.33)		21(10.61)		115(17.97)	73(13.42)		34(19.54)	18(11.25)	
rs3112562	C	562(69.21)	506(71.88)	0.260	210(71.11)	0.606	149(75.25)	0.100	436(68.13)	398(73.16)	0.064	126(73.26)	108(67.50)	0.279
	G	250(30.79)	198(28.13)		86(28.89)		49(24.75)		204(31.88)	146(26.84)		46(26.74)	52(32.50)	

a *χ*^2^-Test or Fisher’s exact test.

The stratification by gender demonstrated that rs9933638, rs12443621, and rs3104746 were significantly associated with lung cancer risk of both male (*P* < 0.001, *P* = 0.003, and *P* = 0.038, respectively) and female subjects (*P* = 0.007, *P* = 0.005, and *P* = 0.049, respectively). Additionally, the analysis of the 16 SNPs among patients with adenocarcinoma and squamous cell carcinoma, the two most common types of NSCLC, respectively, revealed that rs9933638, rs12443621, and rs3104746 were significantly associated with risk of both adenocarcinoma (*P* < 0.001, *P* < 0.001, and *P* = 0.047, respectively) and squamous cell carcinoma (*P* = 0.006, *P* = 0.020, and *P* = 0.008, respectively), while rs3095661 was found to be only related with risk of adenocarcinoma (*P* = 0.009) (Table 2).

### Association of the genotypes of the four SNPs (rs9933638, rs12443621, rs3104746, and rs3095661) with lung cancer risk

As shown in Table 3, consistent with the association between the alleles of the three SNPs (rs9933638, rs12443621, and rs3104746) and lung cancer risk, there was a significantly different distribution of the genotypes of the three SNPs between lung cancer cases and controls (*P* < 0.001, *P* < 0.001, and *P* = 0.003, respectively). The multivariate logistic regression analyses with adjustment of age, gender, and smoking revealed that individuals with rs9933638GG had an elevated risk of lung cancer compared with rs9933638GA and rs9933638AA carriers (codominant model, OR = 2.571, 95%CI = 1.710-3.867, *P* < 0.001, and OR = 1.509, 95%CI = 1.022-2.229, *P* = 0.038, respectively). The dichotomic analysis further demonstrated that individuals with rs9933638GG showed an increased risk of lung cancer compared with rs9933638(GA+AA) carriers (dominant model, OR = 1.877, 95%CI = 1.423-2.476, *P* < 0.001). Additionally, individuals with rs9933638(GG+GA) also displayed an increased risk of lung cancer compared with rs9933638AA carriers (recessive model, OR = 1.893, 95%CI = 1.308-2.741, *P* = 0.001), suggesting that individuals with the allele G of rs9933638 were susceptible to lung cancer in a dose-dependent manner. Similar findings were discovered for rs12443621. For rs3104746, the dichotomic analysis demonstrated that compared with individuals with rs3104746(TA+AA), rs3104746TT carriers had an increased risk of lung cancer (dominant model, OR = 1.665, 95%CI = 1.243-2.230, *P* = 0.001), suggesting that individuals with rs3104746TT were susceptible to lung cancer compared with rs3104746(TA+AA) carriers. Moreover, though a marginally significant difference of rs3095661 alleles was found between controls and cases (*P* = 0.041, Table 2), no significant difference of rs3095661 genotypes was found in the four models between controls and cases (*P* = 0.362, *P* = 0.066, *P* = 0.999, *P* = 0.197, respectively, Table 3). Notably, the detection of rs3095661CC carriers only in lung cancer patients (6/352, 1.70%) but not in controls strongly suggested that rs3095661CC might be risk factor of lung cancer.

**Table 3:** Association of the genotypes of the four SNPs (rs9933638, rs12443621, rs3104746, and rs3095661) at the TOX3/LOC643714 locus with lung cancer risk

SNPs	Model	Genotype	Control (n = 407)	Case (n = 352)	*P*-value^a^	Adjusted *P*-value^b^	OR (95% CI)
rs3095661	Codominant	G/G	321(78.87)	259(73.58)	0.194	0.362	Ref.
		G/C	86(21.13)	87(24.72)	0.008	0.999	0.000(0.000-.)
		C/C	0(0.00)	6(1.70)	0.029	0.999	0.000(0.000-.)
	Dominant	G/G	321(78.87)	259(73.58)	0.103	0.066	0.749(0.550-1.020)
		G/C-C/C	86(21.13)	93(26.42)			
	Recessive	G/G-G/C	407(100.00)	346(98.30)	0.010	0.999	0.000(0.000-.)
		C/C	0(0.00)	6(1.70)			
	Overdominant	G/G-C/C	321(78.87)	265(75.28)	0.260	0.197	0.815(0.597-1.112)
		G/C	86(21.13)	87(24.72)			
rs9933638	Codominant	G/G	117(28.82)	152(43.18)	0.001	0.000	Ref.
		G/A	211(51.97)	161(45.74)	0.000	0.000	2.571(1.710-3.867)
		A/A	78(19.21)	39(11.08)	0.067	0.038	1.509(1.022-2.229)
	Dominant	G/G	117(28.82)	152(43.18)	0.000	0.000	1.877(1.423-2.476)
		G/A-A/A	289(71.18)	200(56.82)			
	Recessive	G/G-G/A	328(80.79)	313(88.92)	0.002	0.001	1.893(1.308-2.741)
		A/A	78(19.21)	39(11.08)			
	Overdominant	G/G-A/A	195(48.03)	191(54.26)	0.094	0.059	1.287(0.991-1.673)
		G/A	211(51.97)	161(45.74)			
rs12443621	Codominant	G/G	130(31.86)	153(43.47)	0.020	0.000	Ref.
		G/A	191(46.81)	153(43.47)	0.000	0.000	2.250(1.512-3.348)
		A/A	87(21.32)	46(13.07)	0.062	0.029	1.534(1.045-2.253)
	Dominant	G/G	130(31.86)	153(43.47)	0.001	0.000	1.646(1.244-2.178)
		G/A-A/A	278(68.14)	199(56.53)			
	Recessive	G/G-G/A	321(78.68)	306(86.93)	0.003	0.001	1.824(1.272-2.616)
		A/A	87(21.32)	46(13.07)			
	Overdominant	G/G-A/A	217(53.19)	199(56.53)	0.381	0.338	1.141(0.871-1.493)
		G/A	191(46.81)	153(43.47)			
rs3104746	Codominant	T/T	266(65.36)	267(75.85)	0.002	0.003	Ref.
		T/A	133(32.68)	79(22.44)	0.788	0.448	1.469(0.544-3.964)
		A/A	8(1.97)	6(1.70)	0.778	0.796	0.875(0.318-2.408)
	Dominant	T/T	266(65.36)	267(75.85)	0.002	0.001	1.665(1.243-2.230)
		T/A-A/A	141(34.64)	85(24.15)			
	Recessive	T/T-T/A	399(98.03)	346(98.30)	1.000	0.635	1.271(0.473-3.417)
		A/A	8(1.97)	6(1.70)			
	Overdominant	T/T-A/A	274(67.32)	273(77.56)	0.002	0.001	1.662(1.234-2.240)
		T/A	133(32.68)	79(22.44)			

a *χ*^2^-Test or Fisher's exact test.

b Adjusted by age, gender, and cigarette smoking.

### Distribution of rs9933638, rs12443621, and rs3104746 among lung cancer patients stratified by cigarette smoking

Because rs9933638, rs12443621, and rs3104746 were found to be associated with lung cancer risk, the distribution of the three SNPs was analysed among lung cancer patients stratified by median number of pack-years of cigarette smoking to investigate whether the gene-environment interaction exists. The linear-by-linear association test revealed that except rs3104746 (*P* = 0.638), the risk allele G of both rs9933638 and rs12443621 showed an increasing trend from light smoking to heavy smoking groups (*P* = 0.004 and *P* < 0.001, respectively, *p* trend < 0.001, Table 4). Multivariate logistic regression analyses adjusted for age and gender revealed that there was a significantly different distribution of genotypes of both rs9933638 and rs12443621 between light smoking patients and heavy smoking patients. Compared with patients with rs9933638GA and 9933638AA, patients with rs9933638GG were enriched and demonstrated an increased risk of lung cancer in heavy smoking subjects (codominant model, OR = 1.714, 95%CI = 1.155-2.542, *P* = 0.007; OR = 2.811, 95%CI = 1.405-5.625, *P* = 0.003, respectively, Table 4). In the dominant model, patients with rs9933638GG carriers were enriched and showed an elevated risk of lung cancer in heavy-smoking patients compared with rs9933638(GA+AA) carriers (OR = 1.878, 95%CI = 1.290-2.736, *P* = 0.001). In the recessive model, lung cancer risk of individuals with rs9933638(GG+GA) in heavy-smoking patients was 2.87 times higher than that of rs9933638AA carriers (OR = 2.868, 95%CI = 1.573-5.227, *P* = 0.001). Similarly, patients with rs12443621GG were also enriched in heavy smoking patients and demonstrated an increase risk of lung cancer compared with rs12443621GA and rs12443621AA carriers (codominant model, OR = 1.821, 95%CI = 1.222-2.713, *P* = 0.003, and OR = 3.067, 95%CI = 1.541-6.104, *P* = 0.001, respectively, Table 4). In the dominant model, individuals with rs12443621GG carriers were enriched in heavy-smoking patients and showed an elevated risk of lung cancer compared with rs12443621(GA+AA) carriers (OR = 2.018, 95%CI = 1.381-2.947, *P* < 0.001). In the recessive model, lung cancer risk of individuals with rs12443621(GG+GA) in heavy-smoking patients was 2.87 times higher than that of rs12443621AA carriers (OR = 2.868, 95%CI = 1.599-5.143, *P* < 0.001).

**Table 4:** Distribution of genotypes of rs9933638, rs12443621, and rs3104746 among lung cancer patients stratified by cigarette smoking

SNPs	Model	Genotype	light-smokings (n = 228)(%)^a^	Heavy-smokings (n = 124)(%)^a^	*P*-value^b^	Adjusted *P*-value^c^	OR (95% CI)
rs9933638	Allele	G	282(61.84)	181(72.98)	0.004		
		A	174(38.16)	67(27.02)			
	Codominant	G/G	86(37.72)	66(53.23)	0.026	0.002	Ref.
		G/A	110(48.25)	49(39.52)	0.012	0.007	1.714(1.155-2.542)
		A/A	32(14.04)	9(7.26)	0.336	0.003	2.811(1.405-5.625)
	Dominant	G/G	86(37.72)	66(53.23)	0.007	0.001	1.878(1.290-2.736)
		G/A-A/A	142(62.28)	58(46.77)			
	Recessive	G/G-G/A	196(85.96)	115(92.74)	0.081	0.001	2.868(1.573-5.227)
		A/A	32(14.04)	9(7.26)			
	Overdominant	G/G-A/A	118(51.75)	75(60.48)	0.119	0.212	1.256(0.878-1.796)
		G/A	110(48.25)	49(39.52)			
rs12443621	Allele	G	282(61.30)	180(73.77)	0.000		
		A	178(38.70)	64(26.23)			
	Codominant	G/G	85(37.28)	66(54.55)	0.013	0.001	Ref.
		G/A	109(47.81)	46(38.02)	0.008	0.003	1.821(1.222-2.713)
		A/A	34(14.91)	9(7.44)	0.259	0.001	3.067(1.541-6.104)
	Dominant	G/G	85(37.28)	66(54.55)	0.002	0.000	2.018(1.381-2.947)
		G/A-A/A	143(62.72)	55(45.45)			
	Recessive	G/G-G/A	194(85.09)	112(92.56)	0.059	0.000	2.868(1.599-5.143)
		A/A	34(14.91)	9(7.44)			
	Overdominant	G/G-A/A	119(52.19)	75(61.98)	0.090	0.176	1.281(0.895-1.832)
		G/A	109(47.81)	46(38.02)			
rs3104746	Allele	T	404(66.01)	58(63.04)	0.638		
		A	208(33.99)	34(36.96)			
	Codominant	T/T	178(78.07)	87(71.90)	0.111	0.140	Ref.
		T/A	45(19.74)	34(28.10)	0.181	0.147	0.645(0.418-0.995)
		A/A	5(2.19)	0(0.00)	1.000	0.999	7.817E8(0.000-.)
	Dominant	T/T	178(78.07)	87(71.90)	0.236	0.140	0.726(0.474-1.111)
		T/A-A/A	50(21.93)	34(28.10)			
	Recessive	T/T-T/A	223(97.81)	121(100.00)	0.168	0.056	7.802(0.953-63.898)
		A/A	5(2.19)	0(0.00)			
	Overdominant	T/T-A/A	183(80.26)	87(71.90)	0.082	0.098	0.697(0.455-1.069)
		T/A	45(19.74)	34(28.10)			

a Lung cancer patients stratified by median number of pack-years of smoking (light smoking, Pack-years of smoking were 0-30; heavy smoking groups, Pack-years of smoking were more than 30).

b *χ*^2^-Test or Fisher's exact test.

c Adjusted by age and gender.

## DISCUSSION

Lung cancer is one of the major causes of cancer-related death worldwide. Besides environmental factors, inherited genetic variants or polymorphisms are also involved in lung cancer risk. In the case-control study, the genotyping of the 16 SNPs at the TOX3/LOC643714 locus in a Han Chinese population revealed that rs9933638/rs12443621 and rs3104746 might contribute to risk of lung cancer. To our best knowledge, the study discovered for the first time that the previously identified breast cancer susceptibility-associated SNPs at the TOX3/LOC643714 locus were risk factors of lung cancer.

The TOX3/LOC643714 locus was one of the first breast cancer regions identified through GWAS in populations of European and East Asian origin [10]. In the recent years, quite a few of SNPs at the TOX3/LOC643714 locus including rs3803662, rs3104746, rs8051542, rs4784227, rs12443621, rs3112612, rs3112562, rs3104793, rs8046994, rs3104788, and rs3104767, were demonstrated to be independently associated with elevated risk of breast cancer and the other human diseases [16–43]. Of the SNPs mentioned above, only rs12443621 and rs3104746 were found to be significantly associated with increased risk of lung cancer in the study. Initially, Pearson's chi-square test or Fisher's exact test revealed that rs9933638 were strongly associated with lung cancer risk (Table 2), confirmed by logistic regression analyses in which lung cancer risk of individuals with rs9933638GG was shown to be 2.57 and 1.51 times higher than that of individuals with rs9933638GA and rs9933638AA, respectively. In addition, lung cancer risk of rs9933638(GG+GA) carriers was demonstrated to be 1.89 times higher than that of rs9933638AA carriers (Table 3). The findings suggested that individuals with the allele G of rs9933638 were susceptible to lung cancer in a dose-dependent manner. To validate the findings, rs12443621, the previously identified breast cancer risk factor of Chinese population [25, 28, 37] and covered by the haplotype block represented by rs9933638, was genotyped in the study. The genotyping data showed that the allele G of rs12443621 was also a risk factor for lung cancer (Table 2), verified by the stratification analysis which revealed that subjects with rs12443621GG and rs12443621GA had an increased risk of lung cancer compared with individuals with rs12443621AA (Table 3). The validation results strongly confirmed that rs9933638, representing a haplotype block covering rs12443621, was associated with risk of lung cancer. Most importantly, rs12443621, the previously determined breast cancer risk factor of Chinese population [25, 28, 37], was discovered to be a lung cancer risk factor of Chinese population in the study provided new evidences that lung cancer and breast cancer are linked at a molecular and genetic level at least in part in Chinese population, which may help to explore the novel carcinogenesis mechanisms.

Rs3803662 was reported to be associated with breast cancer risk of both male and female subjects [13, 16–20]. Consistent with rs3803662, the stratification by gender revealed that all of the three SNPs (rs9933638, rs12443621, and rs3104746) were all associated with increased lung cancer risk of both male and female individuals. Furthermore, the three SNPs were all indicated to be related with elevated lung cancer risk of patients with adenocarcinoma and squamous cell carcinoma. The findings suggested that the association of the three SNPs with lung cancer risk was gender- and histology-independent. Moreover, the analysis of the three SNPs among lung cancer patients stratified by cigarette smoking revealed that except rs3104746, the risk allele G of both rs9933638 and rs12443621 was enriched in lung cancer patients with heavy cigarette smoking and lung cancer risk of individuals with rs9933638/rs12443621(GG+GA) was 2.87 times higher than that of rs9933638/rs12443621AA carriers among heavy smoking patients (Table 4), demonstrating that individuals carrying risk genotypes and with heavy cigarette smoking may have a higher risk of lung cancer. The findings strongly confirmed that the gene-environment interaction exists in the development and process of lung cancer.

There are several limitations in the study. First, due to the fact that most of lung cancer patients recruited were characterized to be poorly differentiated (316/352, 89.77%) and most of patients with NSCLC were identified at advanced stages (276/304, 90.79%), we tried but failed to clarify the association of the 16 SNPs with histological grade and stage of patients with lung cancer. Second, the subjects of the study were recruited only from Southwestern China, large-scale studies are required to clarify the association of the SNPs at the TOX3/LOC643714 locus with lung cancer risk in the other Han Chinese populations.

In summary, the present study discovered for the first time that rs9933638/rs12443621 and rs3104746, the previously identified breast cancer susceptibility-related SNPs at the TOX3/LOC643714 locus, contributed to the individual's risk to lung cancer in the Southwestern Han Chinese population. The findings provided additional evidences that lung cancer and breast cancer are correlated at a molecular and genetic level at least in part in Chinese population.

## MATERIALS AND METHODS

### Study population

Patients (n = 352) with primary lung cancer diagnosed from September 2007 to December 2008 were recruited from the Institute of Human Respiratory Disease of Xinqiao Hospital, the Third Military Medical University. All patients were newly diagnosed, histologically confirmed and previously untreated. 407 age- and sex frequency-matched healthy control samples were collected from individuals at the Centre of Physical Examination of Xinqiao Hospital between November 2007 and December 2008. The exclusion criterion for the control group was any history of cancer. All of the subjects were unrelated at least within three generations. After explaining the purpose and procedures of the study, all participants signed a written informed consent form, completed a detailed questionnaire regarding their smoking habits, and donated 5 ml peripheral blood. Blood samples were drawn into Na-EDTA tubes from all subjects and stored at −70°C for genomic DNA extraction. The study was approved by the Ethical Committee of Xinqiao Hospital, the Third Military Medical University.

### Selection of SNPs

Totally, 16 SNPs at the TOX3/LOC643714 locus were selected in the study. Of the 16 SNPs, six SNPs (rs8051542, rs12443621, rs3803662, rs3104746, rs3112562, and rs4784227) were selected based on the published references in which these SNPs were suggested to be susceptible to breast cancer or the other human diseases and the other 10 SNPs were selected from the genetic variation data for TOX3 gene obtained from the HapMap project for 45 healthy Chinese Han Beijing (CHB) adults (www.hapmap.org). Haplotype blocks, representing regions inherited without substantial recombination in the ancestors of the current population, were constructed throughout the entire TOX3 gene using Haploview (version 4.0, Broad Institute of MIT and Harvard, Cambridge, MA) [45]. The history of recombination between a pair of SNPs can be estimated with the use of the normalized measure of allelic association D' (value of D prime between the two loci) [46, 47]. The criterion for the selected SNPs to construct a haplotype block is that all SNPs in one region must be in strong LD with D' > 0.98 for the upper 95% confidence bound and > 0.7 for the lower bound. A maximally informative htSNP was then selected from each block using the software Tagger program (http://www.broad.mit.edu/mpg/haploview). This algorithm selects a subset of variants that capture all known common genetic variations in the TOX3 gene based on a LD threshold of r^2^ ≥ 0.8. The inverse of r^2^ represents the ratio of sample size needed to detect an indirect association with an un-analyzed SNP to direct association at the same power.

### Genotyping analysis

Genomic DNA was extracted from whole blood using the QIAamp DNA Blood Mini Kit according to the manufacturer's instructions (QIAGEN, Maryland, USA). The SNP genotyping was performed using an improved multiplexligation detection reaction (iMLDR) technique (Genesky Biotechnologies Inc., Shanghai, China). In brief, the selected SNP loci were genotyped in one ligation reaction. Two multiplex PCR reactions were designed to amplify fragments covering all SNP loci. The primer information of the two reaction mixtures is described in Supplementary Table S1 and S2, respectively. The PCR for both reactions was 95°C, 2 min; 11 cycles (94°C, 20s; 65°C −0.5°C/cycle, 40s; 72°C, 1 min 30s); 24 cycles (94°C, 20s; 59°C, 30s; 72°C, 1 min 30s); 72°C, 2 min; hold at 4°C. The ligation cycling programme was 95°C, 2 min; 38 cycles (94°C, 1 min; 56°C, 4 min); hold at 4°C. Half a microlitre of ligation product was loaded into the ABI 3730XL and the raw data were analysed by GeneMapper 4.1.

### Data analyses

Cigarette smoking was stratified by the median number of pack-years of combined cases and controls (1 pack-year = 20 cigarettes per day for 1 year). Cases and controls were compared by Student's *t*-test for continuous variables and Pearson's chi-square test or Fisher's exact test for categorical variables. The Hardy-Weinberg equilibrium of each SNP was tested by SNPStats (http://bioinfo.iconcologia.net/snpstats/start.htm). Each component of the model was: codominant model (major allele homozygotes vs. heterozygotes vs. minor allele homozygotes), dominant model (major allele homozygotes vs. heterozygotes + minor allele homozygotes), recessive model (major allele homozygotes + heterozygotes vs. minor allele homozygotes), and overdominant model (major allele homozygotes + minor allele homozygotes vs. heterozygotes). To assess the independent effect of each SNP, the multivariate logistic regression analyses with adjustments for possible confounding factors (age, gender, and smoking habits) were performed to estimate the association between the SNPs and cancer risk as well as the possible gene-environment interactions. All associations were presented as odds ratios (ORs) with the corresponding 95% confidence intervals (95%CI). All statistical analyses were performed using the Statistical Package for Social Science 15 for Windows (SPSS Inc, Chicago, IL, USA). In the statistical analysis, all statistical tests were two-sided and *P* < 0.05 was considered significant.

## SUPPLEMENTARY TABLES




