# Supplementary material for: The breast cancer susceptibility-related polymorphisms at the TOX3/LOC643714 locus associated with lung cancer risk in a Han Chinese population
Source: Oncotarget. 2016 Jul 28;7(37):59742–53. doi: 10.18632/oncotarget.10874 (PMC5312345; doi:10.18632/oncotarget.10874)
Supplement: Supplementary file 1 [file oncotarget-07-59742-s001.pdf]

The breast cancer susceptibility-related polymorphisms at the TOX3/LOC643714 locus associated with lung cancer risk in a Han Chinese population

SUPPLEMENTARY TABLES

Supplementary Table S1: Primer information of the selected 16 SNPs at the TOX3/LOC643714 locus

		Primer Sequences	Tm	PCR product Size (bp)
rs3095661	F	CACTGGGAAGCTGAATGCATGA	65.69	180
	R	TCGCCAGAGGCATGGTTAAAAA	65.84	
rs10852413	F	CCCCTCTACAAAGGGGGCTTTA	64.55	326
	R	TCAAATGAAAATACCACCCTTTCATTC	64.18	
rs16951204	F	TGTGCCATGATGGATCAAGTGTG	66.33	314
	R	TGTTGAACCTCCAACCTTTGGTGT	65.93	
rs4784219	F	CCAATGCATCAACCACGAACAC	65.70	343
	R	GAAGGGAGGAAATTGGCATCTGA	65.69	
rs9302555	F	TGTGGCATAGGCCAGTAGGTATGA	64.81	152
	R	CGCTTTCACCCACAGCAGGTAG	66.31	
rs8051542	F	TCTGCAGAGACAGGGAGCTCTGATA	65.97	274
	R	TGATGTGCATGGGTTGTACCTG	64.00	
rs9933638	F	GGCTTCCAGAGAATGGCCTCAT	65.99	322
	R	GGCTCAATCAATCCTCCCACCT	65.78	
rs12443621	F	TGACAGAAACCTTGGCTTGGAAAA	66.03	342
	R	AGGCCCCAATAATTTGGAATTTG	64.04	
rs3095604	F	TGAAGCCTCGTGTCGGTTCTCT	65.71	329
	R	AGATCTCACGGCCTCTCCACAA	65.61	
rs1362550 rs28463809 rs4784226	F	TGGCCACAGGAGAGGAGCAG	65.95	609
	R	CAGCTCATCACCCGGATTCTTC	65.33	
rs3803662	F	GGTGGGGGTCAGTCCACAGTTT	66.66	341
	R	TGCTGCTAGTCCTTGGCTGTTC	64.07	
rs4784227	F	GCGGTAAAGGAACCTGGCTGAG	66.34	326
	R	CCAGGCATATGGTCCAGAAAGTTG	65.86	
rs3104746	F	CAGTGGGGAGGGGGTTCTTACA	66.51	324
	R	GATGCCCCTGGGAGCAGAGTAT	65.90	
rs3112562	F	GTCTAGGCAGCTTTGGCCCACT	66.08	220
	R	TGGCTTAGAAATGGCTGGAGAGTG	65.87	

Supplementary Table S2: Primer information of double reaction

SNPs	Primer
rs3095661FG	TTCCGCGTTCGGACTGATAT GCCAGCCAACTCAGTACCAGG
rs3095661FC	TACGGTTATTCGGGCTCCTGT GCCAGCCAACTCAGTACCAGC
rs3095661FP	AGAGAGATATAGAAGAAMCCTGACATAACCA TTT
rs10852413FC	TCTCTCGGGTCAATTCGTCCTT GTGCTGTGTATTTTTATTTGGTAAATCTGGCAAC
rs10852413FT	TGTTCGTGGGCCGGATTAGT GTGCTGTGTATTTTTATTTGGTAAATCTGGCGAT
rs10852413FP	CCTACCATGGTGGTTAAACTARTCAGAA
rs16951204RC	TCTCTCGGGTCAATTCGTCCTT GGTGCTCCAGACCCATCCTCG
rs16951204RG	TGTTCGTGGGCCGGATTAGT GGTGCTCCAGACCCATCCTCC
rs16951204RP	ATAATCATCAGTATTCATCATCATCCGC T
rs4784219RT	TTCCGCGTTCGGACTGATAT GTCATCCAAGGCAATATTCAGAAGGACTA
rs4784219RA	TACGGTTATTCGGGCTCCTGT GTCATCCAAGGCAATATTCAGAAGGACTT
rs4784219RP	AGAAATTTGCAACTCAGTATAGTGAAAGATATTC
rs9302555RG	TTCCGCGTTCGGACTGATAT GGAAGATCTCCCCTACCTGTATGGTACAGC
rs9302555RT	TACGGTTATTCGGGCTCCTGT GGAAGATCTCCCCTACCTGTATGGTACAGA
rs9302555RP	GAATTGTGGTGTAAACAAGCCAATTACT
rs8051542RC	TCTCTCGGGTCAATTCGTCCTT TTTTTGCTCCAATCATAGTGCTTCG
rs8051542RT	TGTTCGTGGGCCGGATTAGT TTTTTGCTCCAATCATAGTGCTCCA
rs8051542RP	TCCTCTAATAACCTAAATGTTTAAAACACATGA
rs9933638RG	TCTCTCGGGTCAATTCGTCCTT GACATGAGCTACTGTACCCAGCCTTCTAGACAC
rs9933638RA	TGTTCGTGGGCCGGATTAGT GACATGAGCTACTGTACCCAGCCTTCTAGACAT
rs9933638RP	TCCTAATTCTTATTGTGACATAAAATTATTAATTAGTTGA
rs12443621FG	TTCCGCGTTCGGACTGATAT CGTTTTATATGCATTAGGCCTGGAAG
rs12443621FA	TACGGTTATTCGGGCTCCTGT CGTTTTATATGCATTAGGCCTGGGAA
rs12443621FP	TGAACTTGAGGTAGGTATTACTATCTCCTTATTTC TTT
rs3095604FG	TTCCGCGTTCGGACTGATAT TCAGCCTTGGCGAAGGGTAG
rs3095604FC	TACGGTTATTCGGGCTCCTGT TCAGCCTTGGCGAAGGGTAC
rs3095604FP	GGGCTTTATACTAAATGGGAACGTTG TTT
rs1362550RC	TCTCTCGGGTCAATTCGTCCTT TGTGCAAAGAAAAACTTAGCTTTCCACAG
rs1362550RG	TGTTCGTGGGCCGGATTAGT TGTGCAAAGAAAAACTTAGCTTTCCACAC
rs1362550RP	TTTTGGCTCCTTGTAAGAGCTATCTG
rs28463809RG	TCTCTCGGGTCAATTCGTCCTT TTTCTAGGCTCTACCGCCCAATGC
rs28463809RT	TGTTCGTGGGCCGGATTAGT TTTCTAGGCTCTACCGCCCAACGA
rs28463809RP	TGAATGCAGTACATCAGCCCAC T
rs4784226RC	TTCCGCGTTCGGACTGATAT AAGACTTTTCCAAAACAATGGCCTGG
rs4784226RT	TACGGTTATTCGGGCTCCTGT AAGACTTTTCCAAAACAATGGCCTGA
rs4784226RP	AGTTAGGGATTGTCACCGAGCA TTTT
rs3803662FG	TTCCGCGTTCGGACTGATAT GGTCAGTCCACAGTTTTATTCTTCGCTATGG
rs3803662FA	TACGGTTATTCGGGCTCCTGT GGTCAGTCCACAGTTTTATTCTTCGCTACGA
rs3803662FP	GACAGCTATAGAGGCATTAAGGAGAGAAA T
rs4784227FC	TCTCTCGGGTCAATTCGTCCTT GGAAAAAAGTCCCAATTTGTAGTGTTTTCC
rs4784227FT	TGTTCGTGGGCCGGATTAGT GGAAAAAAGTCCCAATTTGTAGTGTTTCCT
rs4784227FP	GATTATTGTGATGTAAATACTCCCATCATGA TTT
rs3104746FA	TTCCGCGTTCGGACTGATAT GAAGTCACTGAAGGGACTGTGAGTTAGCA
rs3104746FT	TACGGTTATTCGGGCTCCTGT GAAGTCACTGAAGGGACTGTGAGTTAGCT
rs3104746FP	TTGTGGGCAAMTGGGGTTTC
rs3112562RC	TCTCTCGGGTCAATTCGTCCTT TGTCTGCTGGGGACTTTATATACAAAATGTG
rs3112562RG	TGTTCGTGGGCCGGATTAGT TGTCTGCTGGGGACTTTATATACAAAATGTC
rs3112562RP	ATAATCCATGYGATATTTCTAGGATGATG T

Supplementary Table S3: Basic information and exact test for Hardy-Weinberg equilibrium of the selected 16 SNPs

See Supplementary File 1
