# Supplementary material for: The breast cancer susceptibility-related polymorphisms at the TOX3/LOC643714 locus associated with lung cancer risk in a Han Chinese population
Source: Oncotarget. 2016 Jul 28;7(37):59742–53. doi: 10.18632/oncotarget.10874 (PMC5312345; doi:10.18632/oncotarget.10874)
Supplement: Supplementary file 2 [file oncotarget-07-59742-s002.docx]

**Supplementary Table 3** Basic information and exact test for Hardy-Weinberg equilibrium of the selected 16 SNPs

| SNPs | Chr. Position | SNP Property | Gene Name | MAF (HapMap-HCB) | MAF (the study) | Subject | N11 | N12 | N22 | N1 | N2 | *P* value |
| --- | --- | --- | --- | --- | --- | --- | --- | --- | --- | --- | --- | --- |
| rs3095661 | 52495753 | Intron 4 | TOX3 | 0.153 | 0.127 | All subjects | 580 | 173 | 6 | 1333 | 185 | 0.074 |
|  |  |  |  |  |  | Control | 321 | 86 | 0 | 728 | 86 | 0.017 |
|  |  |  |  |  |  | Lung Cancer | 259 | 87 | 6 | 605 | 99 | 0.672 |
| rs10852413 | 52505145 | Intron 2 | TOX3 | 0.256 | 0.188 | All subjects | 504 | 227 | 27 | 1235 | 281 | 0.818 |
|  |  |  |  |  |  | Control | 274 | 117 | 16 | 665 | 149 | 0.434 |
|  |  |  |  |  |  | Lung Cancer | 230 | 110 | 11 | 570 | 132 | 0.622 |
| rs16951204 | 52507624 | Intron 2 | TOX3 | 0.375 | 0.321 | All subjects | 353 | 315 | 91 | 1021 | 497 | 0.112 |
|  |  |  |  |  |  | Control | 180 | 176 | 51 | 536 | 278 | 0.437 |
|  |  |  |  |  |  | Lung Cancer | 173 | 139 | 40 | 485 | 219 | 0.140 |
| rs4784219 | 52523597 | Intron 2 | TOX3 | 0.263 | 0.250 | All subjects | 399 | 320 | 39 | 1118 | 398 | 0.000 |
|  |  |  |  |  |  | Control | 210 | 175 | 22 | 595 | 219 | 0.060 |
|  |  |  |  |  |  | Lung Cancer | 189 | 145 | 17 | 523 | 179 | 0.102 |
| rs9302555 | 52528847 | Intron 2 | TOX3 | 0.201 | 0.217 | All subjects | 464 | 260 | 36 | 1188 | 332 | 0.956 |
|  |  |  |  |  |  | Control | 239 | 153 | 16 | 631 | 185 | 0.160 |
|  |  |  |  |  |  | Lung Cancer | 225 | 107 | 20 | 557 | 147 | 0.133 |
| rs8051542 | 52534167 | Intron 2 | TOX3 | 0.186 | 0.177 | All subjects | 502 | 230 | 14 | 1234 | 258 | 0.034 |
|  |  |  |  |  |  | Control | 271 | 115 | 8 | 657 | 131 | 0.294 |
|  |  |  |  |  |  | Lung Cancer | 231 | 115 | 6 | 577 | 127 | 0.049 |
| rs9933638 | 52539597 | Intron 2 | TOX3 | 0.444 | 0.391 | All subjects | 269 | 372 | 117 | 910 | 606 | 0.533 |
|  |  |  |  |  |  | Control | 117 | 211 | 78 | 445 | 367 | 0.323 |
|  |  |  |  |  |  | Lung Cancer | 152 | 161 | 39 | 465 | 239 | 0.709 |
| rs12443621 | 52548037 | Intron 2 | TOX3 | 0.390 | 0.398 | All subjects | 283 | 344 | 133 | 910 | 610 | 0.110 |
|  |  |  |  |  |  | Control | 130 | 191 | 87 | 451 | 365 | 0.283 |
|  |  |  |  |  |  | Lung Cancer | 153 | 153 | 46 | 459 | 245 | 0.429 |
| rs3095604 | 52581979 | 5'-flanking | TOX3 | 0.289 | 0.324 | All subjects | 351 | 324 | 84 | 1026 | 492 | 0.479 |
|  |  |  |  |  |  | Control | 188 | 180 | 39 | 556 | 258 | 0.666 |
|  |  |  |  |  |  | Lung Cancer | 163 | 144 | 45 | 470 | 234 | 0.142 |
| rs1362550 | 52582816 | 5'-flanking | TOX3 | 0.185 | 0.190 | All subjects | 508 | 212 | 39 | 1228 | 290 | 0.008 |
|  |  |  |  |  |  | Control | 261 | 130 | 16 | 652 | 162 | 0.970 |
|  |  |  |  |  |  | Lung Cancer | 247 | 82 | 23 | 576 | 128 | 0.000 |
| rs28463809 | 52583054 | 5'-flanking | TOX3 | 0.350 | 0.326 | All subjects | 355 | 316 | 89 | 1026 | 494 | 0.149 |
|  |  |  |  |  |  | Control | 192 | 176 | 40 | 560 | 256 | 0.971 |
|  |  |  |  |  |  | Lung Cancer | 163 | 140 | 49 | 466 | 238 | 0.037 |
| rs4784226 | 52583143 | 5'-flanking | TOX3 | 0.218 | 0.248 | All subjects | 430 | 283 | 46 | 1143 | 375 | 0.950 |
|  |  |  |  |  |  | Control | 241 | 134 | 32 | 616 | 198 | 0.033 |
|  |  |  |  |  |  | Lung Cancer | 189 | 149 | 14 | 527 | 177 | 0.020 |
| rs3803662 | 52586341 | UTR | LOC643714 | 0.347 | 0.326 | All subjects | 357 | 308 | 94 | 1022 | 496 | 0.032 |
|  |  |  |  |  |  | Control | 189 | 172 | 46 | 550 | 264 | 0.471 |
|  |  |  |  |  |  | Lung Cancer | 168 | 136 | 48 | 472 | 232 | 0.018 |
| rs4784227 | 52599188 | Intron 2 | LOC643714 | 0.256 | 0.244 | All subjects | 426 | 294 | 39 | 1146 | 372 | 0.120 |
|  |  |  |  |  |  | Control | 234 | 148 | 25 | 616 | 198 | 0.805 |
|  |  |  |  |  |  | Lung Cancer | 192 | 146 | 14 | 530 | 174 | 0.032 |
| rs3104746 | 52601100 | Intron 2 | LOC643714 | 0.202 | 0.151 | All subjects | 533 | 212 | 14 | 1278 | 240 | 0.175 |
|  |  |  |  |  |  | Control | 266 | 133 | 8 | 665 | 149 | 0.062 |
|  |  |  |  |  |  | Lung Cancer | 267 | 79 | 6 | 613 | 91 | 0.955 |
| rs3112562 | 52608263 | Intron 2 | LOC643714 | 0.367 | 0.284 | All subjects | 384 | 300 | 74 | 1068 | 448 | 0.173 |
|  |  |  |  |  |  | Control | 201 | 160 | 45 | 562 | 250 | 0.129 |
|  |  |  |  |  |  | Lung Cancer | 183 | 140 | 29 | 506 | 198 | 0.761 |
